# Immunopathology of Tumefactive Demyelinating Lesions-From Idiopathic to Drug-Related Cases

**DOI:** 10.3389/fneur.2022.868525

**Published:** 2022-03-15

**Authors:** Aigli G. Vakrakou, Maria-Evgenia Brinia, Ioanna Svolaki, Theodore Argyrakos, Leonidas Stefanis, Constantinos Kilidireas

**Affiliations:** ^1^Demyelinating Diseases Unit, 1st Department of Neurology, School of Medicine, Aeginition Hospital, National and Kapodistrian University of Athens, Athens, Greece; ^2^Department of Pathophysiology, School of Medicine, National and Kapodistrian University of Athens, Athens, Greece; ^3^Department of Pathology, Evaggelismos Hospital, Athens, Greece

**Keywords:** tumefactive multiple sclerosis, brain biopsy, Creutzfeldt–Peters cells, B cells, T cells, granulocytes, MOG-antibody-associated demyelination, NMO (neuromyelitis optica)

## Abstract

Tumefactive demyelinating lesions (TDL) represent a diagnostic dilemma for clinicians, and in rare atypical cases a collaboration of a neuroradiologist, a neurologist, and a neuropathologist is warranted for accurate diagnosis. Recent advances in neuropathology have shown that TDL represent an umbrella under which many different diagnostic entities can be responsible. TDL can emerge not only as part of the spectrum of classic multiple sclerosis (MS) but also can represent an idiopathic monophasic disease, a relapsing disease with recurrent TDL, or could be part of the myelin oligodendrocyte glycoprotein (MOG)- and aquaporin-4 (AQP4)-associated disease. TDL can appear during the MS disease course, and increasingly cases arise showing an association with specific drug interventions. Although TDL share common features with classic MS lesions, they display some unique features, such as extensive and widespread demyelination, massive and intense parenchymal infiltration by macrophages along with lymphocytes (mainly T but also B cells), dystrophic changes in astrocytes, and the presence of Creutzfeldt cells. This article reviews the existent literature regarding the neuropathological findings of tumefactive demyelination in various disease processes to better facilitate the identification of disease signatures. Recent developments in immunopathology of central nervous system disease suggest that specific pathological immune features (type of demyelination, infiltrating cell type distribution, specific astrocyte pathology and complement deposition) can differentiate tumefactive lesions arising as part of MS, MOG-associated disease, and AQP4 antibody-positive neuromyelitis optica spectrum disorder. Lessons from immunopathology will help us not only stratify these lesions in disease entities but also to better organize treatment strategies. Improved advances in tissue biomarkers should pave the way for prompt and accurate diagnosis of TDL leading to better outcomes for patients.

## Key Points

- Tumefactive demyelinating lesions (TDL) are commonly associated with multiple sclerosis (MS) and can emerge during the disease course as part of the spectrum of MS lesions or as the initial presenting radiographic feature or could be either associated with MS drug-related events.
- TDL could also represent an idiopathic monophasic disease, a relapsing disease with recurrent TDL, or could be part of the myelin oligodendrocyte glycoprotein (MOG)- and aquaporin-4 (AQP4)-associated disease.
- TDL display some unique features such as extensive and widespread demyelination, massive and intense parenchymal infiltration by macrophages along with lymphocytes (mainly T but also B cells), dystrophic changes in astrocytes and the presence of Creutzfeldt cells.
- Specific immunopathologic features of TDLs, response to B cell-depletion therapies and exacerbation upon specific MS-related drug manipulations suggest a unique disease entity that requires further investigation.

## Introduction

Tumefactive demyelinating lesion (TDL) is a locally aggressive form of demyelination, defined as a tumor-like lesion <2 cm in the central nervous system (CNS). TDLs can occur as single or multiple lesions and present with mass effect, ring enhancement, and with or without surrounding edema (1). These lesions are often found in the supratentorial region, most commonly in the frontal and parietal lobes, but basal ganglia, infratentorial and spinal cord lesions can also occur. The clinical presentation of patients with TDLs varies owing to differences in size and location of the lesions and the degree of mass effect. Hemiparesis or hemiplegia are the most common clinical manifestations. However, other symptoms, including headache, aphasia, cognitive and visual disturbances, and sensory disorders may also be present (2). The contribution of a neuropathologist is of the utmost importance for diagnosis of TDL that presents atypically or when the diagnosis is not appreciated radiologically. Differential diagnosis includes neoplasms (glial tumors, primary CNS lymphoma), metastasis, brain abscess, granulomatous disease, and vasculitis. New neuroimaging techniques are important to verify the diagnosis, and biopsy can be warranted if imaging isn't conclusive.

### Tumefactive Demyelination Associated With Multiple Sclerosis and Atypical Demyelinating Syndromes

TDL is commonly associated with multiple sclerosis (MS) and can emerge during the disease course as part of the spectrum of MS lesions or as the initial presenting radiographic feature.

Tumefactive MS (TMS) is a rare MS variant that has not been studied in depth due to the lack of large patient cohorts (3). The term TMS in this Review refers to patients with TDLs preceding classical MS or occurring as part of an otherwise typical MS course. Currently, there are no precise clinical/serological and/or radiographical biomarkers assessing the risk of disease evolution and conversion to clinically definite MS. This information would be critically important for clinicians, as it could help determine the therapeutic strategy after the first TDL appearance.

MS variants, such as Marburg's disease, Balo's concentric sclerosis, and Schilder's disease, have also been shown to present with tumefactive lesions, with overlapping clinical presentation, and distinct immunological signatures (4). Nevertheless, it remains controversial whether tumefactive CNS lesions represent a variant of MS or a unique form of an idiopathic isolated demyelinating disease. TDLs are considered a heterogeneous group of demyelinating disorders, extending from an isolated monophasic disease (isolated TDL) to a recurrent form of the disease (recurrent TDL), with or without the presence of classical clinico-radiological MS features (5–7).

TDLs can also occur in patients with other atypical demyelinating syndromes, such as acute disseminated encephalomyelitis (ADEM), aquaporin 4 (AQ4) IgG seropositive or MOG- seropositive Neuromyelitis Optica Spectrum Disorder (NMOSD), as well as other neuroinflammatory disorders including neurosarcoidosis and Behçet's disease (8). Of note, the spectrum of MOG antibody-related encephalomyelitis has been enriched during the last years to include cases with presentation of TDL and ADEM-like manifestations (9, 10). Interestingly, TDLs have been linked to specific disease-modifying drugs (e.g., fingolimod, natalizumab) used in MS, particularly after drug beginning or discontinuation (11–13). The assumption that TDL represents a unique form of isolated atypical demyelinating disease, without classical radiological MS features, presenting either as a monophasic disease (monophasic TDL) or as recurrent TDL attacks (relapses with recurrent TDL), needs further evidence and more studies assessing the underlying molecular and cell-type signatures in these patients (7).

#### Histopathology Findings

The basic histopathologic features of tumefactive demyelination resemble classical MS and indicate an active intense inflammatory demyelinating disease (14). Active TDLs consist of areas of demyelination with relative axonal sparing, inflammatory infiltrates mainly by myelin-containing foamy macrophages, perivascular lymphocytes, and reactive astrocytes that may contain multiple nuclei (Creutzfeldt–Peters cells). Creutzfeldt–Peters cells can also be found in glioblastomas and are usually indicative of rapid and severe demyelination and are seen in certain leukodystrophies. Till nowadays a restricted number of specific biomarkers exist to differentiate among the various subtypes of atypical demyelinating diseases. Recently, it has been found that patients with TDLs present elevated levels of interleukin (IL)-6 in the CSF, similar to those seen in patients with NMOSD and CNS lymphoma, and higher than those in patients with MS. In contrast, CSF IL-10 levels were similar to those of patients with MS and NMO (15).

In our recent study, we analyzed the characteristics and outcomes of patients with recurrent TDLs (16). We also provided a therapeutic algorithm mainly based on the observed clinico-radiological response to treatment options at various time points. We speculate that patients with recurrent TDLs might comprise a distinct group in the inflammatory demyelinating disease spectrum. The pathogenetic mechanisms of TMS are elusive, and further functional and phenotypic experiments are needed to investigate whether such disease comprises a distinct demyelinating disease or a subset of MS with specific radiological features (Table 1).

**Table 1:** Main characteristics of atypical demyelinating lesions and multiple sclerosis (MS) variants.

	**Active MS lesion (acute phase)**	**Tumefactive multiple sclerosis**	**MOGAD**	**NMO (acute phase)**
Inflammation arises:	Focal demyelinating lesions consisting of inflammatory infiltrates, mainly orchestrated around veins and venules that fuse to confluent plaques	Perivenous distribution of lesions described in few cases, perivascular inflammation in general	Small veins and venules, small lesions confluent into larger plaques	Vasculocentric pattern of demyelination
Perivascular inflammation	Macrophages, CD8 T cells (mainly), CD4 T cells, B and plasma cells	Macrophages, CD8 T cells, CD4 T cells (variable), few B cells (in specific cases more prominent)	Macrophages, CD4 T cells (mainly), less CD8 T and B cells	Macrophages, CD4 T cells mainly, less B and plasma cells
Parenchymal infiltration	Macrophages and CD8 T cells mainly	Macrophages, variable levels of lymphocytes (CD8, CD4 T cells, fewer B cells)	Macrophages, CD4 T cells (mainly), less CD8 and B cells	Macrophages, CD4 T cells mainly, less B cells
Myelin phagocytosis	Prominent	Prominent	Moderate (dominant loss of MOG compared with other myelin proteins such as MBP and MAG)	mild (loss of MAG)
Demyelination	Lesions are expanded radially and are prominent in the periventricular space and subcortical white matter. Subpial cortical lesions also a neuropathology hallmark	Extensive, widespread, mainly confluent and less partial demyelination pattern	Confluence of small venules give rise to large plaques of demyelination. Cortical pathology also dominant.	Variable degree of confluent demyelination. Lack of prominent cortical demyelination (in later stages)
Axonal preservation	Partial axonal preservation	Partial axonal preservation, widespread axonal damage might be seen	Partial axonal preservation	Severe axonal loss
Astrocytosis	Reactive astrocytic gliosis	Reactive astrocytic gliosis and in some cases dystrophic changes in astrocytes	Reactive astrocytic gliosis	Astrocyte loss
Creutzfeldt Peter cells	Infrequently	Ocassionally found	Not described	Not described
IgG deposition	Pattern II MS pattern	Limited number of studies: IgG deposits located in the parenchyma with perivascular reinforcement but without clear rosette aspect. In Marburg variant observed in some cases	Diffuse IgG deposition observed and also in lesions without activated complement components	Deposits of IgG and IgM colocalizing with products of complement activation in a vasculocentric pattern around thickened hyalinized blood vessels
Complement deposition	Pattern II MS pattern, mainly complement in macrophages not perivascular	Not assessed in most studies (evidence in some Marburg cases and in rare fulminant tumefactive cases)	In few cases complement in macrophages and perivenous (not so prominent as in NMO). Heterogeneity in studies.	Perivascular complement deposition (characteristic) vasculocentric pattern of complement activa-tion
Patterns of demyelination	I to III (pattern IV very rare)	Pattern I and II described only in limited numbers of cases (Pattern III in cases of Balo's disease)	“Pattern II MS” in a subset of lesions	“MS lesion patterns II and III” in a subset of lesions
Mature oligodendrocyte loss	No (oligodendrocytopathy mainly in pattern-III demyelination, oligodendrocyte loss described in newly forming early MS cases in areas with no evident inflammation)	Partially loss	No (in some cases only)	Yes (MAG loss and distal oligodendrocytopathy also described)
AQP4 loss	No	No	No	Yes
Eosinophils	No	No	No	Yes

*MS, Multiple Sclerosis; MOGAD, Myelin oligodendrocyte glycoprotein associated disease; NMO, neuromyelitis optica; AQP4, aquaporin-4; MAG, myelin associated glycoprotein; MOG, myelin oligodendrocyte glycoprotein; MBP, Myelin basic protein, Patterns I-IV of MS lesion pathology according to Luccinetti et al. 17*

In this article, we aim to review all current knowledge on the immunopathology of TDL with emphasis on biopsy-proven MS-, NMO-, NMOSD-, and MOG-related demyelination. Other variants (e.g., ADEM, acute hemorrhagic encephalomyelitis etc.) are beyond the scope of this review.

## Lessons From the Immunopathology of MS and Other Demyelinating Diseases

### MS

The inflammatory response leading to demyelination in MS is a result of multi-directional feedback involving CNS-resident cells and infiltrating immune cells. In most cases (85–90%), the disease initiates with a relapsing-remitting course (RRMS), which usually develops into a progressive course (secondary progressive MS, SPMS) with ongoing neuroinflammation. For some patients with MS (10–15%), neurological disability increases progressively without relapse or remission (primary progressive MS, PPMS) (18). Findings from animal models and immunological studies in patients with MS indicate that peripheral immune responses targeting the CNS drive disease during the early phases, whereas immune reactions within the CNS dominate the progressive phases. Chronic inflammation, which occurs behind a closed blood–brain barrier with activation of microglia and continued involvement of T and B cells, is a hallmark pathophysiological feature.

Regarding immunopathology, it has been shown that the typical active MS lesions (MS plaques), commonly found in early RRMS, are characterized by focal confluent demyelination that primarily develops around a central vein and consists of diffuse inflammatory infiltration with peripheral macrophages, microglia, T lymphocytes, and some plasma cells (Table 1). The identification of specific intracytoplasmic myelin breakdown products in macrophages could further characterize as theme as actively demyelinating (minor myelin proteins such as MOG, MAG) or post- demyelinating (major myelin proteins such as MBP, PLP) (19). Heterogeneity exits regarding immunopathology of active MS lesions and four distinct patterns (I, II, III, and IV) have been described by Lucchinetti et al. (17). Pattern I, which is that of the “standard” active lesion, is characterized by activated microglia/macrophages (CD68) and T cells centered around veins/venules. Pattern II lesions are distinguished by the evidence of immunoglobulin and complement deposition. Pattern III lesions display a selective loss of myelin-associated glycoprotein (MAG), while oligodendrocyte apoptosis is observed. Pattern IV lesions exhibit a non-apoptotic loss of oligodendrocytes and are mostly observed in patients with PPMS (17).

Patients with progressive MS display the characteristic slowly expanding or smoldering lesions in the white matter (20). Recent studies have shown that CNS-resident microglia are the major myeloid cells present in progressive MS lesions with a preferential accumulation of resident microglia with M1 differentiation at the lesion edge (21–25). Especially in progressive MS, parenchymal or meningeal inflammation may play an important role and has been termed “compartmentalized CNS intrinsic inflammation” (26, 27). Data indicate that immune effector mechanisms associated mostly with progressive forms of MS include clonal expansion of B cells, ectopic formation of follicle-like structures, microglia, and astrocytic activation (26, 28). Recently, meningeal ectopic B cell follicles that could support germinal center activity and plasma cell maturation and survival have been described in SPMS and to a lesser extent in PPMS (29–31).

### NMO-Related Demyelination

Neuromyelitis optica (NMO, formerly known as Devic disease) is an inflammatory antibody- mediated CNS disease that is associated with serological testing of autoantibody marker directed against AQP4, a water channel on astrocytes (AQP4-IgG), and typically presents with optic neuritis, transverse myelitis, and variable, if any, brain involvement. However, in 2007 the term NMOSD was introduced to expand the clinical manifestation of the disease and include patients seropositive for AQP-4 IgG with limited or inaugural forms, including cerebral, diencephalic, and brainstem lesions (32). NMOSD is stratified further as NMOSD with or without AQP4-IgG. TDLs in patients with NMOSD is not a common clinical presentation. Kim et al. (33) described MRI abnormalities in 78 AQP4 seropositive patients and found that 79% had brain lesions on MRI and 29% had extensive hemispheric lesions while 10% had extensive lesions at initial presentation.

Acute NMO lesions are characterized by extensive macrophage/microglia infiltrations, B and T lymphocytes in varying numbers, and a prominent number of granulocytes and eosinophils surrounding the vessels. Main feature of these lesions is the vasculocentric pattern deposition of complement C9neo and IgG (34–36). AQP4 loss in NMO active lesions of the CNS is indicated as a pathologic hallmark feature (34) (Table 1). The demyelination pattern demonstrates heterogeneity depending on the stage of the lesion from myelin preservation to destructive demyelinated lesions. Early NMO lesions can be described as lacking demyelination and without evidence of acute axonal pathology despite AQP4 loss (36, 37). Acute demyelinating lesions show numerous macrophages with myelin degradation products and severe axonal loss (37, 38). Other histopathological features include myelin and tissue vacuolation (35, 39), vascular hyalinization (34, 35), and macrophages containing GFAP positive debris (37). Necrosis and cavitation typically involve both the gray and white matter (40). GFAP is largely lost or decreased within the lesions, and astrocytes are described as dystrophic (37). Brück et al., initially expanded the spectrum of pathology findings among AQP4-IgG-seropositive NMO patients and reported that early NMO lesions showed oligodendrocyte apoptosis associated with a selective loss of MAG (35). Pathological features resembling the II and III patterns of MS including complement activation within macrophages and simultaneous MAG loss and apoptosis of oligodendrocytes, were reported in these patients (35). Astrocyte proliferation has also been reported in such patients (35) (Table 1). In consistency with findings of Bruck et al., the group of Masaki et al., demonstrated that NMO cases demonstrated apart from astrocytopathy (preferential loss of astrocytic Cx43), distal oligodendrogliopathy as well (Cx43/Cx47 astrocyte-oligodendrocyte gap junctions lost), emerging in the active and chronic active stages, but not during the chronic inactive stage of the disease. Misu et al. 1 year later further extended the lesion heterogenicity in active lesions in NMO even in an individual patient basis by introducing six different lesion types. Some important aspects in NMO pathophysiology revealed in this study were findings of either necrosis of astrocytes associated with complement deposition in their plasma membrane, findings of an apoptosis-like process in astrocytes (clasmatodendrosis), which was associated with internalization of AQP4 and AQP1, and astrocyte apoptosis in the absence of complement activation. Another pattern is characterized by clasmatodendrosis of astrocytes, defined by cytoplasmic swelling and vacuolation, beading and dissolution of their processes, and nuclear alterations resembling apoptosis, which was associated with internalization of AQP4 and AQP1 and astrocyte apoptosis in the absence of complement activation. Oligodendrocyte apoptosis and significant loss were found consistently in 4 out of 6 types of lesions (41).

Early reports have suggested that cortical pathology is a common feature in MS but is not present in patients with NMOSDs, when MRI techniques or neuropathology studies were applied (42–44). A more recent study also showed that NMOSD-AQP4 patients showed a relative sparing of deep gray matter volumes and cortical/juxtacortical lesions were seen in none of the NMOSD-AQP4 cases tested (45). Longitudinally extensive spinal cord lesions, commonly seen in NMOSD, involve the central gray matter, where AQP4 is predominantly expressed, compared with the white matter (46, 47). Interestingly, Hayashida et al. (48) described spinal white matter pathology during the early stages of the disease, irrespective of the anti-AQP4 antibody status. In this study, isolated perivascular lesions with selective astrocyte end foot protein loss and not predominating demyelinating features might represent early events in lesion evolution. Additionally, demyelinating type lesions with AQP4 loss with or without complement deposition also reported in this study further expand the heterogeneous NMO pathology in the spinal cord.

### MOG-Related Demyelination

MOG-associated disease (MOGAD) is a rare antibody mediated inflammatory demyelinating disorder of the CNS. MOG is a minor component of myelin and is located at the external lamellae of myelin sheaths and on the surface of oligodendrocytes, which makes it a potential target for autoimmune antibodies and cell-mediated responses (49). MOGAD has been described to manifest with various clinical manifestations including optic neuritis, acute myelitis, NMOSD-like phenotype without AQP4 antibodies, ADEM, MDEM, cortical encephalitis, and, extremely rare, tumefactive-like presentation of demyelination lesions.

Neuropathological reports on MOGAD are scarce. Human pathology studies have shown that most of the MOGAD cases are characterized by perivenous ADEM-like demyelination pattern (50), while fewer present with a transitional pattern with perivenous and confluent white matter demyelination (51). Cortical and intracortical demyelinating lesions are frequent (51). The lesions are infiltrated by abundant phagocytotic myelin-laden macrophages. CD4 T cells dominate compared to CD8 T cells, and fewer CD20 B cells are distributed diffusely in the brain parenchyma and perivascularly. These hypercellular lesions are infiltrated by eosinophils and neutrophils in several cases (51). The oligodendrocytes are well-preserved within the lesions (50). The loss of MOG protein is not selective in all cases. Takai et al. (50) reported 60 of 167 lesions that showed MOG-dominant myelin loss compared to other types of myelin proteins losses. Regarding astrocytes, there is severe astrogliosis and very rare reports of Creutzfeldt Peter cells (51), while the axonal structures are relatively preserved. C9neo complement depositions, especially incorporated by macrophages, but also within the lesions or in a perivenular distribution, have been reported in many cases (50, 51). IgG deposits have rarely been described in the perivenular area of demyelinating lesions (50) and within the cytoplasm of macrophages (52) (Table 1).

## Tumefactive Demyelinated Lesions–Immunopathology

### Immunopathology of TDLs (Idiopathic or in the Spectrum of MS)

**Figure 1:**
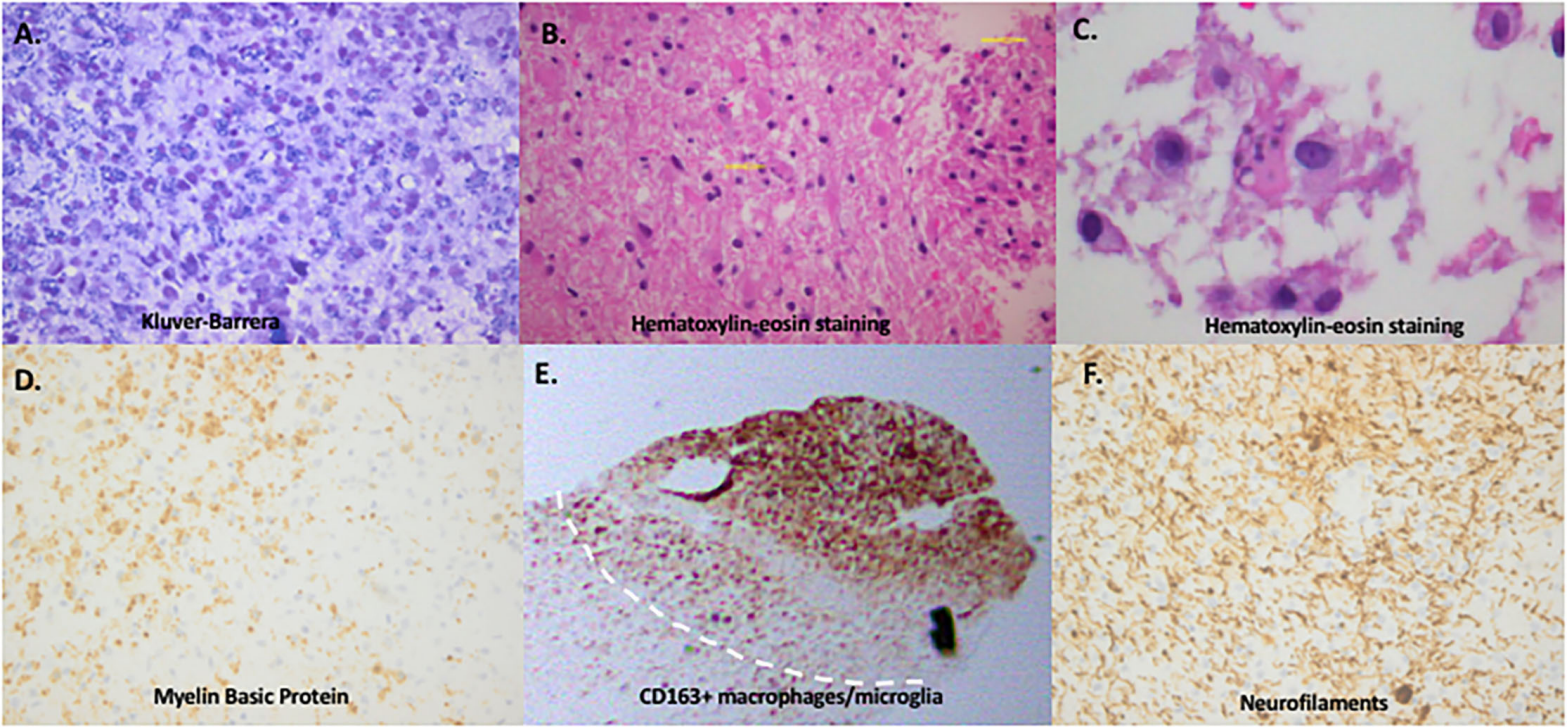
Immunopathology of tumefactive lesions: confluent demyelination, Creutzfeldt-Peters cells and macrophage/microglia-rich area. **(A)** Gradient loss of neuroaxon myelin sheath with evidence of phagocytosis of myelin by the macrophages (Kluver-Barrera histochemical reaction, x400). **(B)** Hematoxylin-eosin staining showing dense infiltratory infiltrates and scattered within the parenchyma giant astrocytes with multiple micronuclei (yellow arrows), also called “Creutzfeldt cells, ” in a brain biopsy of a tumefactive demyelinating lesion. **(C)** Enlarged image of Creutzfeldt-Peters cells (in the center of the image) from the same lesion. **(D)** Loss of myelin sheaths with evidence of phagocytosis by the macrophages (Myelin Basic Protein, x400). **(E)** Mass infiltration by CD163 positive macrophages/microglia cells in a tumefactive demyelinating lesion adjacent to an area of normal appearing white matter. **(F)** Total preservation of neuroaxons (Neurofilaments x400) (images performed and provided by T.A, in Department of Pathology, Evangelismos Hospital, Athens, Greece).

We performed a literature review to summarize the cell subsets involved in the orchestration of cell infiltrates in tumefactive lesions. TDL are characterized by hypercellularity with perivascular and parenchymal lymphocytic infiltrates (1) (Figures 1A–F). Regarding B cells, TDLs are composed of B cells with variable tissue presence ranging from no B cells to rare, few, and intense B cell infiltrates. These are mainly distributed in the perivascular spaces (53, 54). Only few studies have assessed plasma cells in TDLs, and these are mostly described in tumefactive lesions of patients with NMO (53). Plasma cells have been classically detected scattered in the CNS parenchyma of patients with remitting-relapsing and secondary progressive MS, but predominantly at the periphery of B-cell follicles/eLFs in patients with SPMS. CD3 T cells (either CD4 helper or CD8 cytotoxic) are more frequently described to localize around the CNS vessels and to diffusely infiltrate the neural parenchyma (1, 55). One study has compared the infiltrating cell types in patients with TDL (acute phase, biopsies, *n* = 4), in Multiple Sclerosis patients (chronic phase, autopsy, *n* = 11), in MOG-antibody associated disease (*n* = 11, biopsies) and ADEM cases (*n* = 5, biopsies). CD4 and CD8 T cells infiltrating the tissues, as ratios of CD8/CD4 cells, were calculated to be: MOG-disease; 0,7, ADEM; 0,6, TDL; 1,1 and chronic MS; 1,4. These findings show that there is a trend toward more CD4 T cells in MOG and ADEM cases, equal distribution in TDL cases and in MS cases CD8 T cells more consistently and based to previous literature outnumber CD4 T cells (50).

TDLs showed a confluent pattern of demyelination without MOG-dominant myelin loss and a high activity of macrophages phagocytosing myelin debris, especially at the edge of demyelinating plaques (50). The degree of infiltrating B cells expressed as mean cell counts of each cell population/1000 μm^2^ (SD) was 9.6 (11.9) for TDL cases, 4.7 (8.2) for MOG-antibody-associated disease with extensive lesions, 12.4 (12.7) for ADEM cases, 4.0 (23.5) for MS (chronic phase) and 4.1 (6.3) for NMOSD (50). The T/B tissue ratio was 4.9 for TDL cases, 4.9 for MOG-antibody-associated disease, 2.5 for ADEM cases, 2.8 for MS (chronic phase) and 5.8 for NMOSD (50). T cells were the major inflammatory cells in four cases and B cells in three cases, according to another study investigating the inflammatory cell populations in TDL. The CD4/CD8 ratio revealed a prominence of CD8 in most cases (56). In patients diagnosed with atypical idiopathic inflammatory demyelinating lesions with monophasic disease course and different MRI pattern of lesions (infiltrative lesions, ring-like lesion, Baló-like lesion and acute hemorrhagic leukoencephalitis) most of the inflammatory infiltrates were T cells (70% in the total cohort) and fewer B cells (20%) (54). Immunoglobulin G (IgG) deposition has not been assessed in all studies on a routine basis except for the study of Ayrignac et al. (54), in which observed IgG deposits in 61% of the patients; these were located in the parenchyma with perivascular reinforcement, but without a clear rosette feature. In NMO-associated tumefactive lesions, IgG deposition has been described only in a few cases; and there are also cases with no IgG deposition; however, studies with large cohorts are limited (54, 57). Specific C9neo complement deposits incorporated by macrophages have been described only in one case with fulminant TMS (58). Besides, in NMO biopsies, IgG deposits as well as perivascular (vasocentric pattern) and/or parenchymal or even complement deposition in macrophages has more frequently been observed (34). In the study of Takai et al. (50), no complement deposition was found either located perivenularly or within macrophages. Regarding myeloid cells, CD68+ cells represent the major cell type in the lesions. CD68 is a marker not only of macrophages but also of activated microglia. The presence of numerous CD68 cells within the demyelinating lesion that contain myelin degradation products (foamy macrophages) is a very common finding (Figure 1). Data analysis of the microglia phenotype in TDL is missing. Only a few studies have described microglia cells and most of them without assessing their activated or homeostatic status or exact prevalence among infiltrating cells in lesions (53, 54). Interestingly, a new study has shown that activated microglia and macrophages (HLADR positive cells as a marker) form a rim at the lesion borders and show a perivenous accentuation in MOG-positive patients manifesting tumefactive lesions (51). Microglia infiltration occurs also within the cortex that often extends beyond the cortical demyelination (51). The limited number of markers used for microglia identification as well as the overlap with myeloid cell markers of infiltrating macrophages renders such studies difficult to interpret. A common finding of TDLs are reactive astrocytes that may contain multiple nuclei (Creutzfeld–Peters cells) (1, 54). The presence of Creutzfeld–Peters cells may be mistaken for mitotic glial cells (Figure 1). Regarding other histopathology features, TDLs do not frequently display necrotic features or vascular changes (like vessel wall thickening and hyaline changes), and axons are relatively preserved (1, 55) (Table 1). In T2-weighted imaging on MRI, a T2 hypointense rim is present in approximately 15–48% of tumefactive demyelinating lesions (54, 59). It has been shown that myelin degradation and macrophage infiltration account for the T2 low rim of an active MS lesion, whereas ring enhancement is facilitated by the underlying process of angiogenesis and the perivascular macrophage infiltration (60). Regarding TDL, T2-low rim corresponds to peripheral neovascularization and perivascular inflammatory cell infiltration. Endothelial cells of the neovasculature and the infiltrating macrophages in the periphery express vascular endothelial growth factor and monocyte chemoattractant protein-1, which mediate angioneogenesis and inflammation, as indicated my immunohistochemistry experiments (61). Correlation of gadolinium enhancement patterns of tumefactive demyelinating lesions with brain biopsy findings has shown that open-ring and irregular rim patterns of gadolinium enhancement were related to macrophage infiltrations and angiogenesis at the inflammatory lesion border, whereas lymphocytic cuffing accounted for the inhomogeneous pattern of enhancement (62). Necrosis can be rarely seen in cases of severe multiple sclerosis with intense and long-standing inflammation as well as in cases of hemorrhagic leukoencephalopathy (56) (Supplementary Excel Files, Sheet-1, Supplementary References).

### Immunopathology of Marburg's Variant of Multiple Sclerosis

The Marburg's variant of multiple sclerosis, firstly described in 1906, is a fulminant form of MS that rapidly evolves leading sometimes to death or confers severe disability (63). This atypical form of demyelination exhibits some unique characteristics, as lesions are highly destructive with massive macrophage infiltration, prominent axonal injury, and features of tissue necrosis (64).

A study by Suzuki et al. (65) showed well-described autopsy findings from a case of a woman, initially diagnosed with MS that eventually deteriorated and died not responding to first-line treatment with corticosteroids and plasma exchange. The autopsy showed prominent perivascular inflammatory infiltrations in both the parenchyma and meninges. The widespread infiltrates consisted of a mixture of lymphocytes, macrophages, plasma cells, eosinophils, and neutrophils. Interestingly, a peculiar finding, compared to previous rare reports, was the cellular distribution of inflammatory cells; CD20+ B-cells (69.0%) outnumbered CD3+ T-cells (25.0%), CD8+ cytotoxic T-cells (4.1%) and CD4+ helper T-cells (1.1%). The demyelination was evident both in white and gray matter. Axonal injury was evident and correlated with the extent of inflammation and demyelination, whereas oligodendrocytes were relatively intact. Giant reactive astrocytes were also noticed, whereas no deposition of immunoglobulin or complement occurred. Therefore, this case illustrates that tumefactive demyelinating lesions with neuropathology evidence of Marburg disease (extensive demyelination, destructive lesions) could also present with meningeal inflammation and gray matter lesions with inflammatory infiltrate consisted of mainly B-cell accompanied by a Th2 related cytokine signature in the CSF. Rare neuropathology findings of Marburg variant of MS are also presented in Supplementary Tables.

### Immunopathology of Baló's Concentric Sclerosis

Baló's concentric sclerosis (BCS) is a rare demyelinating disease and whether BCS is a distinct demyelinating disorder or a variant of MS or a subtype of tumefactive demyelination is not totally clear. BCS and tumefactive demyelinating lesions are considered to be shared common immunopathogenesis (66). Hardy et al., presented an interesting case with coexisting Baló lesion and a tumefactive demyelinating lesion without evidence of other more typical demyelinating lesions (67). BCS can occur in patients with otherwise typical MS (68). Clinical work from research group recently pointed to clinical heterogeneity subgrouping patients in those that share common features with MS (OCBs, MS-type lesions, relapses, humoral responses) and those with a more aggressive disease requiring higher immunosuppression that might be characterized by a different underlying pathology (68).

First described in 1928, the hallmark of the demyelinating disease Baló concentric sclerosis (BCS) is the alternating demyelinating rings. An important role in concentric demyelinating lesions of Baló disease play the upregulation of hypoxia-induced tissue preconditioning and pro-inflammatory cytokines derived from glial cells. Stadelmann et al., identified the deregulated pathway of hypoxia-inducible factor-1α (HIF-1α) at the periphery of BCS lesions expressed by oligodendrocytes, but also astrocytes (69). Interestingly, the preserved myelin is attributed to tissue-preconditioned oligodendrocytes resistance to further damage at the edge of radially expanding lesions that survived hypoxia-like injury due to unidentified neuroprotective proteins. Also, astrocyte pathology is evident as hypertrophic astrocytes, GFAP-positive exist in both demyelinated and myelin-preserved areas and are closely associated with oligodendrocytes (70). Takai et al. (71) showed that hypertrophic astrocytes express CC motif chemokine 2 and interleukin-1β that further promote demyelination. Extensive loss of AQP4, along with loss of various connexins (Cx43, Cx32, and Cx47), important for astrocyte-oligodendrocyte interactions, in astrocytes without the characteristic of NMO perivascular deposition of immunoglobulin or activated complement has been described suggesting an underlying astrocytopathy as a contributor of Balo disease pathology (72–74). Importantly, most acute lesions from Baló's cases display a homogeneous pathology, not always relevant in MS and MMO cases (75).

Novel neuropathological data regarding the immunopathology of Balo was recently provided by (76). The authors presented 10 concentric lesions from four autopsied Baló's disease cases. Three cases displayed the classical features of distal oligodendrogliopathy (DO), characterizing Baló disease, with preferential loss of myelin-associated glycoprotein (MAG) and preservation of MOG accompanied by activated microglia expressing TMEM119 and glucose transporter 5 (GLUT5) (both markers for microglia), but not the homeostatic microglia marker purinergic receptor P2Y12 (P2RY12). This pattern of activation is associated with the apoptosis of oligodendrocytes in the leading edge of Baló's concentric lesions. In the same study, in the fourth autopsy case with late active demyelination without DO, TMEM119-, GLUT5- and P2RY12-positive microglia with ramified morphology were associated with myelin preservation in concentric lesions, a finding pointing to the role of anti-inflammatory innate immunity subsets as triggers of remyelination.

### Tumefactive Lesions in the Spectrum of NMO and NMOSD Related Demyelination

Although there are many neuropathological studies on brain and spinal cord lesions in patients with NMOSD, there are only few biopsy studies regarding tumefactive presentation in NMO. Here, we systematically searched the biopsied tumefactive NMO cases. The cases were characterized as tumefactive or extensive/large cerebral lesions based on MRI brain scan. The lesions from tumefactive cases that we reviewed were characterized by numerous myelin-laden macrophages diffusely distributed at the brain parenchyma and often perivascular. Inflammatory cells including T or B cells and rarely plasma cells have been found to distribute around the vessels in several cases (77–81). Myelin vacuolation has been described in six cases, which is a typical NMO histopathological feature (77). There were reports on granulocytes, mainly eosinophils, in nine cases (77, 78, 80, 82). They were distributed perivascularly and were present occasionally or as extensive accumulations (78, 80, 82). A common finding of the lesions was the diffuse loss of AQP-4 immunoreactivity, and the loss of GFAP staining implying the reduced number of astrocytes in the lesions. On the other hand, Massey et al. (78) described a patient with clinically definite NMOSD with preservation of astrocytes and AQP4 expression. There were also cases with macrophage infiltration containing GFAP + debris (77). Variable numbers of reactive astrocytes were reported, and in a few cases, astrocytes were described as dystrophic and with degeneration of foot processes (77, 78). In six biopsies increased immunoreactivity of AQP4 immunostaining has been reported in the PPWM (77). Blood vessel thickening and hyalinization have also been reported in eight cases (77, 81, 83). Loss of oligodendrocytes has been indicated in only one case (84). IgG depositions have been described in only one case (78) distributed in a widespread fashion within the brain parenchyma. Specific C9neo complement depositions have been reported in two cases with a vasculocentric pattern (79) and incorporated within the macrophages (84). Popescu et al. (77) described deposition of complement products in a perivascular rim or rosette pattern in eight AQP4 loss biopsies.

Pathology studies of AQP4 seronegative NMO patients are extremely rare. We identified two cases that presented with large brain lesions that were seronegative both for AQP4 and MOG antibodies (54, 85). The lesions were characterized by extensive myelin loss without perivenous distribution, preservation of AQP4 immunostaining, and vascular hyalinization. They were infiltrated by numerous macrophages and B cells and mild numbers of T cell, but there were no accumulations of granulocytes. A varied astrocyte morphology has been described with reactive astrogliosis, dystrophic astrocytes, and Creutzfeldt Peter cells (Supplementary Excel Files, Sheet-2, Supplementary References).

### TDLs in the Spectrum of MOG-Related Demyelination

A systematic literature search identified a limited number of pathology studies of MOGAD cases who presented with large demyelinating lesions. All patients were positive for MOG antibodies confirmed by cell-based assays and their diagnoses varied, including ADEM-like presentation (50, 86), tumefactive MS-like lesions (87–89), NMOSD-like phenotype (90, 91), and large white matter lesions with MOG+ antibodies (92). Less than 10 cases found in the literature strictly presented with TDLs. Brain biopsies of these cases showed hypercellular lesions with relative axonal preservation and reactive astrocytes. The patterns of demyelination, as those seen in active MS lesions, were mainly consisting of MS pattern II lesions, overlapping sometimes with pattern I (88, 92) or III (86). Severe myelin loss that resembles the confluent pattern of demyelination has been described within the lesions, and only one case with severe perivenous demyelination has been reported (89). All lesions were infiltrated by abundant activated macrophages within the brain parenchyma and peri-vascularly, while MOG+ myelin degradation products within the cytoplasm of macrophages were a characteristic finding in some of them (3/10 cases). In only one case activated macrophages have been described at the active lesion edge (90). CD68 and CD163 staining used for the activated macrophages are markers also for activated microglia. However, analysis for the microglia phenotype within the lesions is incomplete. T cells, CD4 helper or CD8 cytotoxic, have been described in all cases to accumulate in the perivascular space and infiltrate diffusely the brain parenchyma in varying numbers. We cannot infer a dominant type of T-cell population among cases. Infiltrations by a moderate number of eosinophils in the perivascular space has been reported in only one case (89). There is great variability regarding B cell infiltrates among the cases, ranging from complete absence (90) to few (86, 88, 91) and mild (92) infiltrates. These cells are mainly present in the perivascular space but also within the parenchyma. Plasma cells were not detected in any of the cases. A common finding was the loss of immunostaining for MOG protein within the lesions. Information on the expression of other myelin proteins, such as MAG (86) or PLP (90), has been found in only two cases, therefore whether there is selective loss of MOG protein among the cases is not certain. In 6 out of 10 cases depositions of activated complement (antigen C9neo) have been described to distributed within the lesion (87–89), on fibers (86, 90), and incorporated by macrophages (86, 87, 90). IgG depositions have been reported in only two cases distributed within the lesion and on the fibrillar structures (86, 90). Finally, one of the larger studies of Höftberger et al. (51) involved in depth analysis of the immunopathology of MOG-related demyelination and included 22 biopsies and two autopsies from patients with MOG antibody associated NMOSD. The one autopsy in this study involved a patient with tumefactive lesion. Postmortem examination of entire brain showed widespread areas of demyelination in the cerebral white matter characterized by multiple perivenous lesions giving rise to confluent inflammatory demyelinating areas. Macrophage cytoplasm showed MOG-positive myelin degradation products. Interestingly, some of the confluent demyelinating lesions showed a rim of activated macrophages and microglia with early active demyelinating activity at the border, like chronic active lesions in classical MS. Even though, relative preservation of axons was observed, axonal spheroids were numerous particularly at the edge of the lesions. One of the most important findings of this outstanding study was the identification of perivenous demyelination in the cortex (small intracortical and single subpial lesions) and in deep gray matter nuclei. Deposition of activated complement (C9neo antigen) was observed at active demyelination within the lesion (51) (Supplementary Excel Files, Sheet-3, Supplementary References).

### Pathology of Drug Induced TDLs

Disease-modifying drugs (e.g., fingolimod, natalizumab) used in MS have been associated with the occurrence of TDLs, especially after drug initiation or cessation. A rebound syndrome can occur as an increase in disease activity after treatment cessation in MS, especially in case of natalizumab or fingolimod. Cases with biopsy-proven TDL development have been described in MS patients under fingolimod treatment (93–96). In all three well-described cases, the predominant perivascular T cell subtype was CD8 cytotoxic T cells, whereas B cells were few. Creutzfeldt cells have been described in one case (93), and in another case deposits of complement and immunoglobulins were identified in the lesion as an antibody/complement mediated type of MS, described previously as MS pattern II (95). An interested case presented by Hashimoto et al. (97) showed the reappearance of a TDL shortly after fingolimod initiation in a woman with a past history of a demyelinating disease beginning with isolated TDL, converted to classical relapsing remitting MS and subsequent new TDL emergence after fingolimod (no biopsy available) (97). There are various hypotheses on TDL emergence in patients with MS after fingolimod initiation. First, TDL appearance has been hypothesized to be potentiated in selected patients by activated CD8^+^ effector T cells that infiltrate CSF (CD45R0negCCR7neg; 2-fold enriched in the CSF vs. blood) and released great amounts of perforin (98). Apart from this exaggerated arm of the adaptive immune response, TDL may emerge as part of the overt activation of the innate immune system as illustrated by the predominant macrophage activation imposed by a proinflammatory milieu (3, 12, 99).

**Figure 2:**
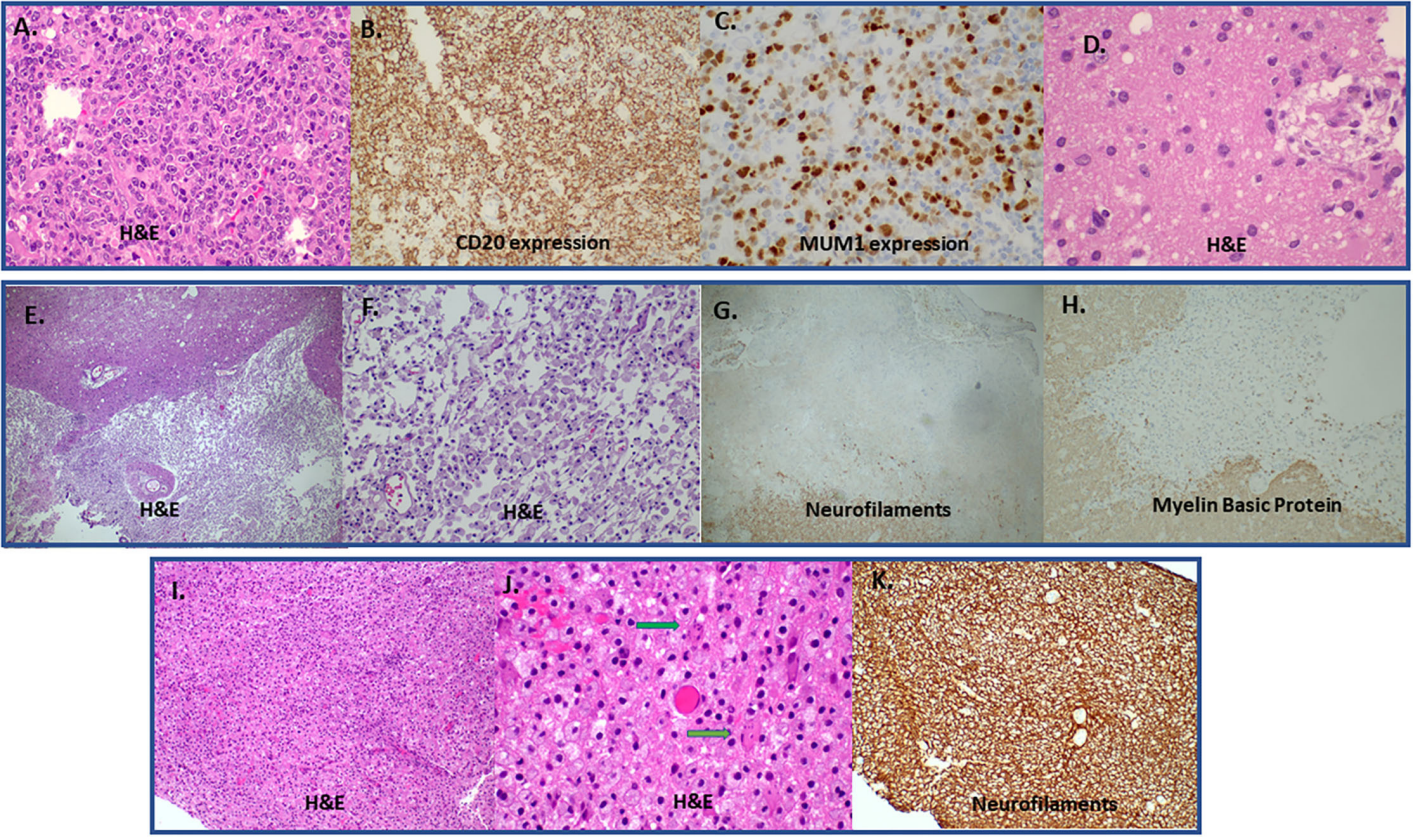
**(A–C)** A case of Diffuse large B-cell lymphoma (DLBCL) that presented with a tumefactive lesion. Lesion with high cellularity and infiltration of brain parenchyma by large B cells **(A)**. Strong CD20 **(B)** and Multiple Myeloma Oncogene 1 (MUM1) protein expression **(C)** by large B cells. There is also an area of regression with perivascular cuff of macrophages (hematoxiln-eosin staining; H&E) **(D)**. **(E–H)** Immunopathology features from a biopsy of a lesion derived from brain ischemic infract initially considered as Glioblastoma multiforme in a young patient. H&E (magnification x100) stained area showing a sharply demarcated macrophage rich lesion **(E)**. Greater magnification (H&, magnification x200) reveals discohesive macrophage infiltration with loosened intercellular connections **(F)**. There is complete absence of neuraxons (total axonal loss) in the macrophage rich area characteristic of an infarct (Neurofilaments, magnification x100) **(G)**. There is also absence of myelin sheaths in the area of axonal loss with concominant absence of myelin granules in the cytoplasm of macrophages (absence of myelinophagia) [**(H)**; Myelin Basic Protein, magnification x200]. **(I–K)** Rebound after fingolimod cessation with TDL emergence. Tightly packed histiocytes in MS after withdrawal of fingolimod [**(I)**; H&E; manification x100]. Typical granular mitosis [**(J)**; Creutzfeldt-Peter cells, arrows (H&E; magnification x400]. Complete preservation of underlying neuraxons [**(K)**; Neurofilaments, magnification x200]. DLBCL, Diffuse large B-cell lymphoma; H&E, hematoxiln-eosin staining; TDL, tumrfactive demyelinating lesion; MS, Multiple Sclerosis.

Fingolimod cessation has also been associated with TDLs (11, 100) (Figure 2). In terms of pathophysiology, fingolimod withdrawal has been linked to pathology of astrocytes with dysregulated sphingosine-1- phosphate signaling (strong immunoreactivity on astrocytes) and prominent astrocytic gliosis.

Sphingosine-1-phosphate signaling is widely expressed in innate immune system cells, but their reaction after fingolimod discontinuation is unknown (11). In a mice model of EAE fingolimod discontinuation led to S1P1 overexpression in lymph node resident lymphocytes leading to immense egress from lymph nodes to CNS causing a severe encephalitogenic attack. Impairment of T-reg cell function also contributed in the loss of peripheral tolerance control facilitated the rebound effect (101). Emergence of TDLs in natalizumab treated patients have been exceptionally reported in the literature, with some cases resulting in death and associated with the detection of anti- natalizumab antibodies. Two cases with TDLs under natalizumab exhibited pattern II of demyelination (102, 103). In one patient with withdrawal and restart of natalizumab multiple tumefactive lesions with a B-cell dominated inflammation were observed (104). Häusler et al. (105) presented first time immunopathological evidence of immune cell alterations in brain of natalizumab treated MS patients (both biopsies and autopsies). They found that immune cells enter the CNS and there is accumulation of plasma cells, whereas a concomitant reduction in dendritic cells. The pathogenic and clinical significance of such immune cell architecture in cases with tumefactive lesions is currently unknown (105).

TDL emergence has been reported on an ocrelizumab-treated patient with Relapsing- Remitting MS and biopsy showed signs of demyelination along with reactive astrocytosis, dense macrophage infiltrates and perivascular lymphocytes (106). This case deserves special attention as the occurrence of TDL under B cell lymphopenia (CD19 = 0.7% lymphocytes) is of particular interest. Authors suggest that ocrelizumab could not halt the disease evolution in this case with fulminant MS, and a more intense immunosuppression with anti-CD20 along with methotrexate could be a prompt therapeutic option, as they did. Follow up data are missing. They suggest that intense CD20 B-cell suppression (pre-infusion serum CD19 = 0) is more appropriate for patients with particularly severe inflammatory disease. We agree with authors, but we also suggest a more in-depth analysis of peripheral immune system during disease exacerbation (B regulatory IL-10 producing cells versus B effectors, B-cell activating factor / BAFF levels), along with signs of active CNS compartmentalized inflammation difficult to be targeted. Moreover, from a clinical perspective, this case teaches us that caution should be taken when ocrelizumab is administered during an ongoing disease activity, as in this case the patient 1 day before scheduled infusion, reported exacerbated weakness, incoordination, and difficulty concentrating.

Interestingly there is one case report of an RRMS patients who developed a tumefactive demyelinating lesion only 4 months after treatment with alemtuzumab. As known, alemtuzumab therapy, an anti-CD52 antibody induces a rapid decrease of lymphocytes the first months that is followed by an early and disproportionate B-cell reconstruction with a later increase in T-lymphocytes. B-cell reconstitution in the absence of peripheral tolerance mechanisms mediated by specific T cells (regulatory T cell subset) has been hypothesized to contribute to the pathogenesis of the TDL in this patient. Apart from the role of B cells, immense infiltration of monocytes in the CNS but absence of immunoreactivity of lymphocyte markers CD4 and CD20 has been described in tissue biopsy of an NMO patient after administration of alemtuzumab (107). Barton et al. (108) presented a patient with typical RRMS who developed a new appearing tumefactive demyelinating lesion (TDL) 4-months after treatment with alemtuzumab with incomplete ring of restricted diffusion and gadolinium enhancement. The most interesting about this case is the analysis of peripheral blood during the emergence of tumefactive demyelination. Lymphocyte subpopulation analysis showed that CD19 B-cell count was increased and composed almost of naïve B cells, whereas CD4 and CD8 T-cell counts were suppressed. This does not explain totally the generation of tumefactive lesion as most patients under alemtuzumab do not exhibit exacerbation with tumefactive features. Nevertheless, this case illustrates that under conditions of T cell lymphopenia, exaggerated B cell responses could lead to disease exacerbation, when an unidentified, either genetic or environmental factor, triggers the immune system to overreact (108).

Tumefactive inflammatory demyelinating lesions as an atypical manifestation of tacrolimus neurotoxicity showed marked hypercellularity and a diffuse presence of infiltrating mononuclear cells with finely vacuolated cytoplasm Numerous macrophages, CD3 T cells, and a few B CD79a cells have been described in the lesion, but the mechanisms leading to such inflammatory infiltrates are obscure (109). Multifocal tumefactive lesions under etanercept showed perivascular hypercellularity with T lymphocytes and macrophages (110). It is well-known the association of TNFa blockers and demyelination and possible explanations include the enhancement of antigen presenting cell function, the uncontrolled T-cell receptor signaling, the reduction of autoreactive T- cells apoptosis, and blockage of TNFR2 receptor involved in maintenance of immune tolerance, remyelination, and oligodendrocyte regeneration (111–113). Nevertheless, how inhibition of TNF can lead to escape of immune surveillance is elusive. Moreover, TNFa related demyelination is debatable if it an inflammatory disease of the white matter, or a primary inflammatory demyelinating disease.

Till today we don't know whether drug induced TDL are triggered by disturbed peripheral cell populations and dynamic cross-talks among cell subset affected by specific drugs or TDL represent an exaggerated activation of brain tissue immune or resident cells provoked by the altered microenvironment imposed by drugs. It seems that an outside-inside model could better explain the drug associated TDLs and more studies focusing on the peripheral immune deregulation (balances in pro-/anti-inflammatory cell signatures, peripheral tolerance mechanisms) could give valuable information. Toward this, literature search revealed a case of a previously healthy man who developed tumefactive MS and peripheral immune cell profiling revealed naïve lymphopenia (low CD4 and CD8 levels) (114) (Supplementary Excel Files, Sheet-4, Supplementary References).

One major limitation regarding interpretation of drug induced tumefactive demyelinating lesions involve the possibility of a rebound disease. Further research should clearly separate idiopathic TDLs which are truly induced by drugs or are part of an exacerbated pathology not corresponding to treatment.

## Red Flags in Differential Diagnosis of Tumefactive Demyelination

Tumefactive lesions are very often mistaken on neuroimaging studies with tumors or abscesses. The most important differential diagnosis for neuropathologists are astrocytoma, oligodendroglioma, glioblastoma, lymphocytic vasculitis, acute disseminated encephalomyelitis, histiocytosis and steroid-responsive CNS lymphoma (8) (Figure 2). Tumefactive demyelinating lesions could be mistaken with astrocytoma or oligodendroglioma due to hypercellularity, but the typical perivascular macrophages with foamy cytoplasm are missing. Vessel wall pathology (distortion or destruction) should always be assessed to rule out an underlying vasculitis. In very rare cases, Primary central nervous system lymphomas (PCNSL) initially present with steroid-responsive, multifocal demyelinating “sentinel” lesions that histologically are characterized by a predominance of T-cell infiltrates (non-neoplastic CD3 cells) and few B-cells (115, 116). It should be stressed that these demyelinating brain lesions may be histologically indistinguishable from those observed in classic demyelinating lesions such as in MS, a notion that can be misleading, especially if corticosteroid administration is preceded (117). Red flags of demyelination preceding the diagnosis of steroid-responsive Central Nervous System Lymphoma involve the disruption of the background neuropil (also called “empty bed”) and the presence of acute apoptosis post steroids. This highlights the importance of repeated biopsies and/or radiological re-examinations in difficult cases (118). Molecular techniques emphasizing clonality of lymphocytes such as BCR sequencing, analysis of immunoglobulin gene rearrangements in CSF or in biopsy specimens, could better facilitate the diagnosis of lymphoma. Regarding possible liquid biomarkers, CSF sIL-2R and IL-10 levels have been suggested to differentiate CNS lymphoma from CNS inflammatory diseases (higher levels in lymphoma) (119). Moreover, CXCL13 and IL-10 have also been proposed in diagnostic algorithms for the workup of brain lesions in which lymphoma is a possible diagnosis (120).

Other TDL mimickers are Progressive multifocal leukoencephalopathy in which demyelination ranges from myelin pallor to severe loss. Virally infected cells (mainly oligodendrocytes) are positive for SV-40 virus and express high levels of Ki67 and p53 (121). In Langerhans cell histiocytosis (LCH) there is accumulation of lymphocytes and histiocytes (myeloid cells with diverse macrophage or dendritic cell phenotypes) that express CD1a, Langerin (CD207), S100 and Cyclin D1 (122). Pathology of Non-Langerhans histiocytosis such as Erdheim-Chester disease involves characteristic Touton giant cells that are CD68 positive and CD1a negative. Rosai- Dorfman disease is characterized by emperipolesis, which is the engulfment by macrophages of intact leukocytes. The macrophages are CD68 positive, S-100 protein positive, and CD1a negative (123). Histiocytic damage can also be observed in the chronic phase of an infract but in such cases there are completely missing axons (cavitation) and macrophages do not engulf myelin (Figure 2).

ADEM/AHLE or acute disseminated encephalomyelitis/acute hemorrhagic leukoencephalitis is a severe variant of ADEM (usually affects children) which manifests with a hyperacute clinical attack. Histopathologically it is characterized by peri venous inflammation and multiple lesions involving gray matter. The identification of hemorrhage on MRI is a distinguishing factor considered to be associated with poor prognosis (124). A histopathological hallmark is the hemorrhagic foci with fibrinoid necrosis of vessel walls as well as the neutrophilic infiltrates. T cells can also be found (54).

An important question in the differential diagnosis of tumefactive lesions is whether the observed demyelinating lesions could be encountered as part of a paraneoplastic syndrome or as part of an autoimmune encephalitis (90, 125, 126). Screening for an underlying malignancy with whole body computed tomography and onconeural antibodies could help in very rare cases such as anti-Ma2– seropositive autoimmune encephalitis in a patient with mediastinal non-seminomatous germ cell tumor (127). Particularly anti-NMDAR encephalitis has been recently associated with demyelinating events with large demyelinating lesions as concurrent or independent episodes (52, 128, 129). Nevertheless, it is currently unknown whether demyelination in these antibody- associated diseases is a specific primary demyelination process or a secondary phenomenon to an unidentified inflammatory mediator.

## Limitations

One limitation of our review is to consider the differences between the autopsies and the biopsies. Autopsies can provide generally a more complete picture of the nature and the extent of cell infiltration, demyelination, and neurodegeneration in comparison to biopsies but the latter can perform on acute attack or exacerbation of CNS pathology and give useful information of early stages of the CNS injury.

## Scientific Questions and Future Directions

In conclusion, this review describes the immunopatterns in TDLs and provides essential evidence for underlying disease-dependent heterogeneity, suggesting the mechanisms and targets of tissue injury in TDLs differ among patient subgroups. Although TMS lesions share common features with classic MS lesion, they display some unique features that should prompt further investigation. The extensive and widespread demyelination, massive and intense parenchymal infiltration by macrophages along with lymphocytes (mainly T, but also B cells), dystrophic changes in astrocytes, and presence of Creutzfeldt cells are some common features encountered in biopsies form patients with TMS. As described, TDLs are associated with MS, but they can occur in neuromyelitis optica spectrum disorders, and testing for AQP4-IgG and MOG-IgG is recommended. Recent developments in immunopathology of CNS disease, suggest that specific pathological immune features (type of demyelination, infiltrating cell type distribution, specific astrocyte pathology and complement deposition) can differentiate tumefactive lesions arising as part of MS, MOG-associated disease and AQP4 antibody-positive NMOSD. It is not clear why tumefactive demyelinating lesions compared to classical MS display astrocytic pleomorphism with a large number of Creutzfeldt–Peters cells and higher cellularity compared to classic non-tumefactive MS lesions. Monophasic tumefactive demyelinating lesions (not fulfilling MS criteria) on the one hand and recurrent TDL (tumefactive lesions that relapse as tumefactive lesions in the same or in different regions) on the other, are disease entities not fully investigated; there is a lack of tissue biopsy biomarkers for differentiating them from classical MS, but there are distinct immunopathology patterns when compared to MOG and AQP4 associated disease. Tissue biomarkers are useful not only in stratifying demyelinating patterns in disease groups but also help in better organizing therapeutic strategies. There is emerging evidence favoring induction strategies over escalation therapies in case of tumefactive demyelinating disorders. B-cell-directed therapies have been shown by us and others to be highly effective especially in cases with recurrent TDLs.

Cyclophosphamide has shown effectiveness in aggressive disease courses indicated by a poor response to corticosteroids and plasma exchange failure. Lessons from drug-related TDL, immunopathology studies of biopsied lesions and clinical cases reports indicate different cellular and humoral mechanisms implicated in the pathogenesis; lymphopenia-induced lymphoproliferation, persistent selected peripheral lymphopenia (CD8 or CD4 T cells), exaggerated innate immunity response either with pathogenic potential or regulatory / compensatory potential and exaggerated B cells response (16, 99).

The pathogenetic mechanisms of tumefactive demyelinating disease remain elusive, and further functional and phenotypic investigations are needed to assess whether such disease represents a distinct demyelinating disorder or a subset of MS with specific radiological features.

## Author Contributions

AV, TA, and CK contributed to conception and design of the study. AV, M-EB, and IS organized the database. AV and M-EB wrote the first draft of the manuscript. IS, TA, and LS wrote sections of the manuscript. All authors contributed to manuscript revision, read, and approved the submitted version.

## Conflict of Interest

The authors declare that the research was conducted in the absence of any commercial or financial relationships that could be construed as a potential conflict of interest.

## Publisher's Note

All claims expressed in this article are solely those of the authors and do not necessarily represent those of their affiliated organizations, or those of the publisher, the editors and the reviewers. Any product that may be evaluated in this article, or claim that may be made by its manufacturer, is not guaranteed or endorsed by the publisher.
