# Supplementary material for: Immunopathology of Tumefactive Demyelinating Lesions-From Idiopathic to Drug-Related Cases
Source: Front Neurol. 2022 Mar 15;13:868525. doi: 10.3389/fneur.2022.868525 (PMC8997292; doi:10.3389/fneur.2022.868525)
Supplement: Supplementary file 1 [file Table_1.DOCX]

**References in the Supplementary Files**

Alderson L, Fetell MR, Sisti M, Hochberg FH, Cohen M, Louis DN. Sentinel lesions of primary CNS lymphoma. J Neurol Neurosurg Psychiatry. 1996;60(1):102–5.

A Moreno-Estébanez, J M Losada Domingo, T González-Pinto González , A Rodríguez-Antigüedad Zarrantz. Isolated pseudotumoural demyelination: A focal, monophasic autoimmune encephalitis? Nurologia (Engl Ed). 2020 Apr;35(3):217-219.

Anderson RCE, Connolly ES Jr, Komotar RJ, Mack WJ, McKhann GM, Van Orman CB, et al. Clinicopathological review: tumefactive demyelination in a 12-year-old girl. Neurosurgery. 2005;56(5):1051-7.

Anders Svenningsson, Ann M Dring, Anna Fogdell-Hahn, Iwan Jones, Elin Engdahl, Malin Lundkvist, Thomas Brännström, Jonathan D Gilthorpe Fatal neuroinflammation in a case of multiple sclerosis with anti-natalizumab antibodies. Neurology. 2013 Mar 5;80(10):965-7.

Bolcaen J, Acou M, Mertens K, Hallaert G, Van den Broecke C, Achten E, Goethals I. Structural and metabolic features of two different variants of multiple sclerosis: a PET/MRI study. J Neuroimaging. 2013;23(3):431-6.

Brandão E, Melo-Pires M, Veira C. Relapsing-remitting tumefactive demyelination. JAMA Neurol. 2014;71(3):366-7.

Cianfoni A, Niku S, Imbesi SG. Metabolite findings in tumefactive demyelinating lesions utilizing short echo time proton magnetic resonance spectroscopy. AJNR Am J Neuroradiol. 2007;28(2):272-7.

Conforti R, Capasso R, Galasso R, Cirillo M, Taglialatela G, Galasso L. A challenging diagnosis of late-onset tumefactive multiple sclerosis associated to cervicodorsal syringomyelia: doubtful CT, MRI, and bioptic findings: Case report and literature review. Medicine (Baltimore). 2016;95(36):e4585.

Cuoco JA, Guilliams EL, Rogers CM, Entwistle JJ, Olasunkanmi AL. Multicentric benign demyelinating pseudotumor: The great masquerade. Neurohospitalist. 2020;10(4):326-8.

DiFrancesco JC, Isimbaldi G, Bedeschi MF, Castellotti B. Biopsy-proven multiple sclerosis in an adult patient with atypical craniometaphyseal dysplasia. BMJ Case Rep. 2018;2018:bcr2017223390.

Fan X, Mahta A, De Jager PL, Kesari S. Rituximab for tumefactive inflammatory demyelination: a case report. Clin Neurol Neurosurg. 2012;114(10):1326-8.

Di Pauli F, Höftberger R, Reindl M, Beer R, Rhomberg P, Schanda K, et al. Fulminant demyelinating encephalomyelitis: Insights from antibody studies and neuropathology. Neurol Neuroimmunol Neuroinflamm. 2015;2(6):e175.

Gnanapavan S, Jaunmuktane Z, Baruteau KP, Gnanasambandam S, Schmierer K. A rare presentation of atypical demyelination: tumefactive multiple sclerosis causing Gerstmann’s syndrome. BMC Neurol. 2014;14:68.

Gupta K, Vasishta RK, Kharbanda PS, Vyas S, Prabhakar, S. Marburg’s disease: a diagnostic dilemma. Neurol Sci. 2011;32(6):1195-201.

Villarreal JV, Abraham MJ, Acevedo JAG, Rai PK, Thottempudi N, Fang X, et al. Tumefactive multiple sclerosis (TMS): A case series of this challenging variant of MS. Mult Scler Relat Disord. 2021;48:102699.

Katsuse K, Kurihara M, Sugiyama Y, Kodama S, Takahashi M, Momose T, et al. Aphasic status epilepticus preceding tumefactive left hemisphere lesion in anti-MOG antibody associated disease Mult Scler Relat Disord. 2019;27:91-4.

Kearney H, Price T, Cryan J, Beausang A, Looby S, Brett FM., et al. Acute multiple sclerosis lesion pathology does not predict subsequent clinical course—a biopsy study. Ir J Med Sci. 2019;188(4):1427-34.

Kimura N, Kumamoto T, Hanaoka T, Hasama Y, Nakamura K, Okazaki, T. Monofocal large inflammatory demyelinating lesion, mimicking brain glioma. Clin Neurol Neurosurg. 2009;111(3):296-9.

Kiriyama T, Kataoka H, Taoka T, Tonomura Y, Terashima M, Morikawa M, et al. Characteristic neuroimaging in patients with tumefactive demyelinating lesions exceeding 30 mm. J Neuroimaging. 2011;21(2):e69-77.

Krivickas LS, Hochberg FH, Freeman S. Chronic inflammatory demyelinating polyradiculoneuropathy with tumefactive central demyelination. Muscle Nerve. 2006;33(2):283-8.

Kuan YC, Wang KC, Yuan WH, Tsai CP. Tumefactive multiple sclerosis in Taiwan. PLoS One. 2013;8(7):e69919.

Letournel F, Cassereau J, Scherer-Gagou C, Bernard I, Mercat A, Gray F, et al. An autopsy case of acute multiple sclerosis (Marburg's type) during pregnancy. Clin Neurol Neurosurg. 2008;110(5):514-7.

Lin M, Reid P, Bakhsheshian J. Tumefactive multiple sclerosis masquerading as high grade glioma. World Neurosurg. 2018;112:37-8.

Ludwig A, Duvall J, Peterson JE, Hakimi R. Marburg’s variant of multiple sclerosis with extensive brain lesions: An autopsy case report. Int J Neurol Neurother. 2015;2(1):027.

Maia C, Novo A, Sousa M, Brás P, Brito O, Rebelo O, et al. Tumefactive demyelinating lesions spectrum disorders and the potential role of contemporary disease modifying treatments: a case report. Mult Scler Relat Disord. 2021;47:102669.

Malhotra HS, Jain KK, Agarwal A, Singh MK, Yadav SK, Husain M, et al. Characterization of tumefactive demyelinating lesions using MR imaging and in-vivo proton MR spectroscopy. Mult Scler. 2009;15(2):193-203.

Mandrioli J, Ficarra G, Callari G, Sola P, Merelli E. Monofocal acute large demyelinating lesion mimicking brain glioma. Neurol Sci. 2004;25 Suppl 4:s386-8.

de Medeiros FC, de Albuquerque LA, Pittella JE, de Souza RB, Gomes Neto AP, Christo PP. Open-ring enhancement in pseudotumoral multiple sclerosis: important radiological aspect. Case reports in neurological medicine, 2014, 2014.

Miyamoto N, Kagohashi M, Nishioka K, Fujishima K, Kitada T, Tomita Y, et al. An autopsy case of Schilder's variant of multiple sclerosis (Schilder's disease). Eur Neurol. 2006;55(2):103-7.

Nagappa M, Taly AB, Sinha S, Bharath RD, Mahadevan A, Bindu PS, et al. Tumefactive demyelination: clinical, imaging and follow‐up observations in thirty‐nine patients. Acta Neurol Scand. 2013;128(1):39-47.

Nakamura M, Endo M, Murakami K, Konno H, Fujihara K, Itoyama Y. An autopsied case of neuromyelitis optica with a large cavitary cerebral lesion. Mult Scler J. 2005;11(6):735-8.

Nakamura M, Itani K, Miyake K, Kunieda T, Kaneko S, Kusaka H. Natalizumab is effective for the treatment of relapsing-remitting tumefactive multiple sclerosis. Intern Med. 2017;56(2):211-4.

Nunes JC, Radbruch H, Walz R, Lin K, Stenzel W, Prokop S, et al. The most fulminant course of the Marburg variant of multiple sclerosis—autopsy findings. Mult Scler. 2015;21(4):485-7.

Ogura H, Fujioka S, Mishima T, Tsugawa J, Fukae J, Tsuboi Y, et al. Comparison of pathology in MS and NMO with tumefactive demyelinating lesions. J Neurol Sci. 2017;381:792.

Omerhodžić I, Džurlić A, Lisica D, Mahmutbegović N, Nikšić M, Bilalović N, Suljić E. Relapsing Tumefactive demyelination: A case report. Acta Med Acad. 2018;47(2):193-8.

Oreja-Guevara C, Gómez-Pinedo U, García-López J, Sánchez-Sánchez R, Valverde-Moyano R, Rabano-Gutierrez A, et al. Inhibition of neurogenesis in a case of Marburg variant multiple sclerosis. Mult Scler Relat Disord. 2017;18:71-6.

Ramos AB, Peters CW, Landry-Wegener B, Cannizzaro LA, Lovera J. A case of neuromyelitis optica spectrum disorder presenting with undiagnosed Sjogren's syndrome and a single, atypical tumefactive lesion: A clinical conundrum. J Neurol Sci. 2017;383:216-8.

Salunke P, Aggarwal A, Gupta K, Agrawal P, Ahuja CK, Vasishta RK. Large demyelinating lesions: A neurosurgical perspective. Br J Neurosurg. 2012;26(4):490-8.

Sánchez JJG, Nora JE, de Notaris M, Arboix JR, García CGA, Rodríguez EF. A case of malignant monophasic multiple sclerosis (Marburg's disease type) successfully treated with decompressive hemicraniectomy. J Neurol Neurosurg Psychiatry. 2010;81(9):1056-7.

Sempere AP, Feliu-Rey E, Sanchez-Perez R, Nieto-Navarro J. Rituximab for tumefactive demyelination refractory to corticosteroids and plasma exchange. J Neurol Neurosurg Psychiatry. 2013;84(12):1338-9.

Siffrin V, Müller-Forell W, von Pein H, Zipp F. How to treat tumefactive demyelinating disease? Mult Scler. 2014;20(5):631-3.

Siri A, Carra-Dalliere C, Ayrignac X, Pelletier J, Audoin B, Pittion-Vouyovitch S, Labauge P. Isolated tumefactive demyelinating lesions: diagnosis and long-term evolution of 16 patients in a multicentric study. J Neurol. 2015;262(7):1637-45.

Štourač P, Kolčava J, Keřkovský M, Kopřivová T, Křen L, Bednařík J. Progressive tumefactive demyelination as the only result of extensive diagnostic work-up: A case report. Front Neurol. 2021;12: 701663.

Suzuki M, Kawasaki H, Masaki K, Suzuki SO, Terada T, Tsuchida T, Miyajima H. An autopsy case of the Marburg variant of multiple sclerosis (acute multiple sclerosis). Intern Med. 2013;52(16):1825-32.

Uehara T, Beck G, Baba K, Mihara M, Okuno T, Sumi H, Mochizuki H. Tumefactive brain lesion with rapid cavity formation associated with anti-aquaporin-4 antibody. Neurol Neuroimmunol Neuroinflamm. 2016;3(3): e230.

Uematsu Y, Fukai J, Okita R, Owai Y, Fujita K, Tanaka Y, Itakura T. Intra-axial pseudotumors in the central nervous system: clinicopathological analysis. Brain Tumor Pathol. 2010;27(2):71-80.

Villa A, Quarracino C, Colobraro A. Pseudotumoral lesion: pathology and follow-up. Acta Neurol Belg. 2016;116:627-8.

Wattamwar PR, Baheti NN, Kesavadas C, Nair M, Radhakrishnan A. Evolution and long term outcome in patients presenting with large demyelinating lesions as their first clinical event. J Neurol Sci. 2010;297(1-2):29-35.

Yamada S, Yamada SM, Nakaguchi H, Murakami M, Hoya K, Matsuno A, Ishida Y. Tumefactive multiple sclerosis requiring emergent biopsy and histological investigation to confirm the diagnosis: a case report. J Med Case Reports. 2012;6:104.

Tomizawa Y, Nakamura R, Hoshino Y, Sasaki F, Nakajima S, Kawajiri S, et al. Tumefactive demyelinating brain lesions with multiple closed-ring enhancement in the course of neuromyelitis optica J Neurol Sci. 2016;361:49-51.

Zaheer K, Ajmeri AN, Singh M, Suliman MS, Teka S. Tumefactive multiple sclerosis, a rare variant presenting as multiple ring-enhancing lesions in an immunocompetent patient: a case report. Cureus 2018;10(12):e3738.
